# Overlap MCAP-CLAPO syndrome associated with malignant ovarian tumor in a 11 year old girl

**DOI:** 10.1016/j.jdcr.2025.09.042

**Published:** 2025-10-14

**Authors:** David Del Cerro Rodríguez, Jesús del Pozo Losada, Felipe Sacristán Lista, Samuel González Pola, Manuel Gómez Tellado

**Affiliations:** a Department of Pediatric Surgery, Universitary Hospital of A Coruña, A Coruña, Spain; b Department of Dermatology, Universitary Hospital of A Coruña, A Coruña, Spain; c Department of Pathology, Universitary Hospital of A Coruña, A Coruña, Spain

**Keywords:** ovarian tumor, PROS

## Introduction

PROS encompasses a group of entities with shared characteristics, primarily presenting with focal or segmental overgrowth. These entities are classified based on clinical manifestations and the main affected organs or tissues. Among these entities we can find capillary malformation of the lower lip, lymphatic malformation, asymmetry, and partial/generalized overgrowth syndrome (MCAP)^1^ and capillary malformation of the lower lip, lymphatic malformation, asymmetry, and partial/generalized overgrowth syndrome (CLAPO).^2^

Depending on the location or type of malformation, various syndromes within the spectrum are identified. These include vascular malformations, distal limb anomalies, renal malformations, or neurodevelopmental disorders.^3^ Despite efforts to classify patients into specific syndromes, the combination of phenotypes often leads to some patients being classified as overlapping entities.^4^

Another classical manifestation associated with this group of patients is the risk of developing certain types of tumors, mainly benign. Although there are very few reported cases of malignant tumors, they are mostly limited to Wilms tumor and nephroblastomatosis. Only sporadic cases of leukemia, vestibular schwannoma, and retinoblastoma have been reported.^5^^,^^6^ The necessity of routine imaging for early detection remains a controversial topic.

## Case report

**Fig 1:**
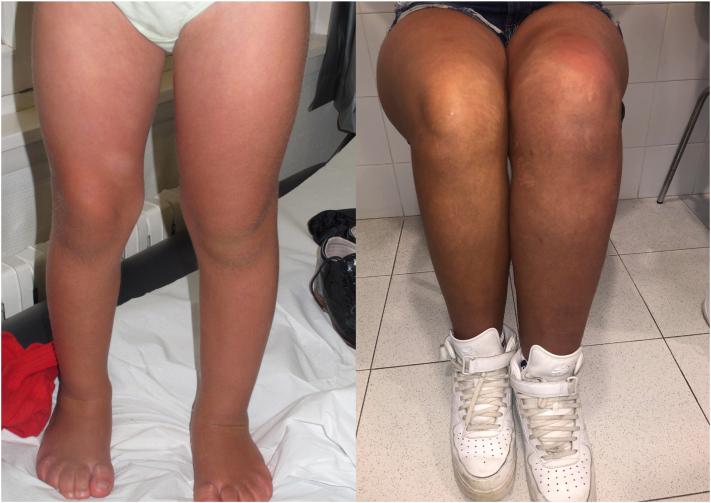
Progressive hemihypertrophy of the left lower limb.

**Fig 2:**
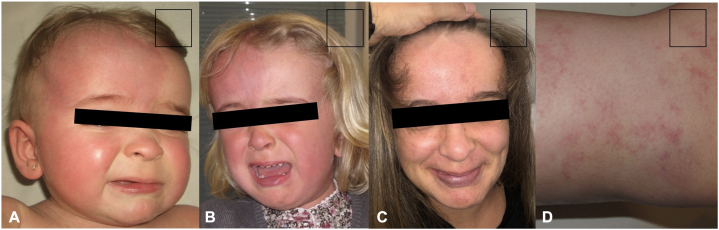
**A** and **B,** Right hemifacial capillary malformation and macrocephaly. **C,** Clearance of hemifacial capillary malformation and increase in intensity of capillary malformation in the lower lip. **D,** Diffuse capillary malformation with a reticulated appearance on the trunk and extremities.

We present a female patient under multidisciplinary follow-up since birth due to macrocephaly, hemihypertrophy of the left lower limb (Fig 1), and an extensive capillary malformation. The capillary malformations exhibit a reticulated appearance across her entire body and face (Fig 2, *A*). It was particularly localized on the right and median side of the face (Fig 2, *B*).

A cerebral magnetic resonance imaging (MRI) at birth revealed mild ventricular dilation and slight left plagiocephaly, leading to a diagnosis of Chiari malformation type 1. Early on, she was diagnosed with attention-deficit hyperactivity disorder (ADHD) at 8 years old.

**Fig 3:**
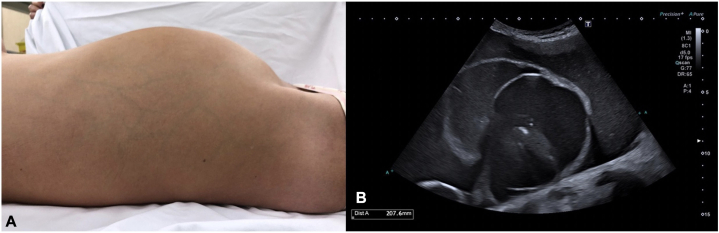
**A,** Abdomen of the patient when referred to the oncohematology unit. **B,** Abdominal ultrasound findings with a solid-cystic mass that occupied almost the entire pelvis and extended to the upper abdomen.

**Fig 4:**
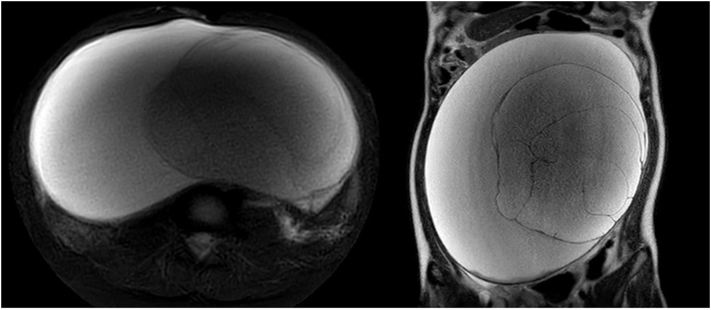
MRI findings. Predominantly cystic abdominal mass measuring 29 × 26 × 12 cm, originating from the left ovary.

At 11 years old, she was referred to oncology for an ultrasound finding of an abdominopelvic cystic mass after progressive asymptomatic abdominal distension over 1 month (Fig 3). Blood tests showed negative tumor markers. An MRI revealed a large solid-cystic mass (29 × 26 × 12 cm), likely originating from the left adnexa (Fig 4).

Histopathological analysis identified it as a borderline mucinous tumor with intraepithelial carcinoma foci. Staging surgery showed no tumor implants.

Immunohistochemical analysis showed positivity for CA19.9, CEA, CSX2, and PTEN protein. Blood genetic testing, including a panel for vascular anomaly-related genes, was negative. Genetic analysis of a vascular lesion biopsy was negative for PTEN but positive for PIK3CA (p.E970K).

Considering the clinical manifestations, our patient presents an overlap phenotype between MCAP and CLAPO syndromes.

The patient has been followed for 4 years post-surgery and remains disease-free.

## Discussion

PROS patients experience significant morbidity, necessitating long-term follow-up by specialized healthcare teams. Given the variability in anatomical, biological, and genetic presentations, a multidisciplinary approach is essential to address potential complications throughout their lives.

This is the first reported case of an ovarian malignant tumor associated with the PROS spectrum. These patients have a relative risk of developing tumors, most of which are benign.

Malignant tumors are much rarer. Among 12 reported cases of Wilms tumors or nephroblastomatosis in patients with PROS, only 2 were MCAP cases, with none in CLAPO patients. The remaining cases include 8 patients with congenital lipomatous overgrowth, vascular malformations, epidermal nevi, scoliosis/skeletal (CLOVES) and 2 with Klippel-Trenaunay syndrome (KTS). These data suggest a 1.4% to 3.3% frequency for Wilms tumor development.^3^ No other solid malignant tumors have been associated with MCAP or CLAPO, and no ovarian malignancies have been reported in PROS patients until now.

However, the genetic PICK3CA variant of our patient has been previously described in 4 articles, 3 of them associated with malignant tumors.^7^, ^8^, ^9^

Some authors recommend abdominal ultrasound screening every 3 months until the age of 8, when tumor risk decreases. However, this is controversial due to the low incidence of malignant tumors, with a 3% risk considered sufficient to justify screening.^3^ The age cutoff of 8 is based on the risk of renal tumors, as the median age of Wilms tumor diagnosis in PROS patients is 27.4 months.^3^ However, our patient’s ovarian tumor at age 11 challenges this cutoff.

This case emphasizes 2 key points: raising awareness about these rare syndromes and highlighting the importance of imaging in patient follow-up for early complication detection. Nevertheless, longitudinal studies are needed to determine the necessity of routine long-term imaging in patients with PROS.

## Conflicts of interest

None disclosed.
